# Estimating the variation in need for community-based social care by body mass index in England and associated cost: population-based cross-sectional study

**DOI:** 10.1186/s12889-017-4665-1

**Published:** 2017-08-22

**Authors:** Vicky R. Copley, Nick Cavill, Jane Wolstenholme, Richard Fordham, Harry Rutter

**Affiliations:** 1 Risk Factors Intelligence, Public Health England, 4150 Chancellor Court, Oxford Business Park South, Oxford, OX4 2GX UK; 2 Cavill Associates, Bramhall, Stockport, Cheshire, SK7 1DQ UK; 3 Health Economics Research Centre, Nuffield Department of Population Health, University of Oxford, Oxford, OX3 7LF UK; 4 Norwich Medical School, Faculty of Medicine and Health Sciences, University of East Anglia, Norwich, Norfolk, NR4 7TJ UK; 5 London School of Hygiene and Tropical Medicine, WC1E 7HT, London, UK

**Keywords:** Social care, BMI, Obesity, Public health, Economics

## Abstract

**Background:**

Adult obesity is linked to a greater need for social care because of its association with the development of long term conditions and because obese adults can have physical and social difficulties which inhibit daily living. Obesity thus has considerable social care cost implications but the magnitude of these costs is currently unknown. This paper outlines an approach to estimating obesity-related social care costs in adults aged over 65 in England.

**Methods:**

We used univariable and multivariable logistic regression models to investigate the relation between the self-reported need for social care and potential determinants, including body mass index (BMI), using data from Health Survey for England. We combined these modelled estimates of need for social care with the mean hours of help received, conditional on receiving any help, to calculate the expected hours of social care received per adult by BMI.

**Results:**

BMI is positively associated with self-reported need for social care. A one unit (ie 1 kg/m^2^) increase in BMI is on average associated with a 5% increase in the odds of need for help with social care (odds ratio 1.05, 95% CI 1.04 to 1.07) in an unadjusted model. Adjusting for long term illness and sociodemographic characteristics we estimate the annual cost of local authority funded care for those who receive it is £599 at a BMI of 23 but £1086 at a BMI of 40.

**Conclusion:**

BMI is positively associated with self-reported need for social care after adjustment for sociodemographic factors and limiting long term illness. The increase in need for care with BMI gives rise to additional costs in social care provision which should be borne in mind when calculating the cost-effectiveness of interventions aimed at reducing obesity.

**Electronic supplementary material:**

The online version of this article (doi:10.1186/s12889-017-4665-1) contains supplementary material, which is available to authorized users.

## Background

The adverse health consequences associated with obesity in adults are well documented. They include type 2 diabetes, cardiovascular disease, liver disease, respiratory disease, musculoskeletal disorders and certain cancers [1]. The association of obesity with the development of long term conditions means that obesity is linked to a greater need for social care. In addition obese adults may have physical and social difficulties which inhibit daily living and can also require social care [2]. Obesity thus has considerable health and social care cost implications [3–8]. These costs, coupled with the current high prevalence of obesity in the general population (25% of adults in England [9]) highlight a requirement for effective interventions targeted at obesity prevention and reduction [10]. Combining estimates of the clinical effectiveness of such interventions with an assessment of their economic impact should help to maximise the efficient allocation of public health resources.

Local authorities in England have had responsibility for public health service commissioning since April 2013 [11]. Public Health England (PHE) is a national body which supports local authorities in delivery of their public health objectives and as part of this remit has developed a cost-effectiveness tool for weight loss interventions that is targeted at local authorities [12]. The first phase of the tool included the costs to the health service of selected comorbidities associated with obesity but did not reflect the wider social costs of obesity because of a lack of available evidence with which to calculate them robustly. The potential economic benefits to a local authority of obesity-reducing interventions were thus underestimated. Local authorities are responsible for social care provision and are perceived to face a considerable cost burden from obesity-related social care costs but there are few published estimates of social care resource use or cost related to BMI levels. Previous studies have been conducted in the older adult populations of Ireland [8] and Europe [13]. There is also a lack of published data that directly link obesity with social care need [2] or enable a calculation of obesity-related social care costs.

The aims of this study were to estimate the impact of BMI on the need for social care in a sample of the English population; and to estimate the expected hours of care, and thereby costs, associated with this level of need. Our findings will inform estimates of the cost-effectiveness of interventions aimed at reducing obesity in local authority settings and elsewhere.

## Methods

### Data

We used data from the Health Survey for England (HSE), an annual survey designed to be representative of the population living in private households in England [14]. The survey uses a multi-stage stratified random probability sample of households; people living in institutions are outside its scope. Since 2011 the survey has included questions for people aged 65 and over about their need for social care, and receipt of such care. Around 63% of people who receive community-based social care in England are aged 65 and over [15]. The surveys do not include information about use of social care in adults aged less than 65.

Social care involves provision of help with personal care and domestic tasks to help people live as independently as possible. The need for and receipt of social care is measured in HSE using self-assessment of a number of Activities of Daily Living (ADLs) and Instrumental Activities of Daily Living (IADLs) (Table 1). ADLs relate to personal care and mobility about the home, while IADLs are additional activities which are important for living independently [15]. For each ADL and IADL participants are asked whether they can carry out the activity on their own; manage on their own with difficulty; only do the activity with help; or not at all.

**Table 1:** Tasks included in HSE Activities of Daily Living (ADLs) and Instrumental Activities of Daily Living (IADLs)

ADLs	IADLs
Getting up and down stairs	Shopping for food
Having a bath or a shower	Doing routine housework or laundry
Dressing or undressing	Getting out of the house
Getting in and out of bed	Doing paperwork or paying bills
Getting around indoors	
Taking medicine	
Using the toilet	
Eating, including cutting up food	
Washing face and hands	

HSE also contains questions on help received in the last month by ADL and IADL, and hours of help received by source in the last week [14]. Sources of help may be formal or informal. We adopt the HSE definitions of informal care but use a more restricted definition of formal care in order better to highlight care which is funded by a local authority. Informal sources of care include partners, family members and friends. Our definition of formal social care includes care provided by: home care workers/home helps/personal assistants; members of the reablement/intermediate care staff team; wardens/sheltered housing managers; and council maintenance workers. It excludes help provided by occupational therapists/physiotherapists; voluntary helpers; and cleaners.

Measured height and weight are obtained during the HSE interviewer visit. Measurements are not taken from participants who are unable to stand or are unsteady on their feet and weight measurements are not taken from participants who are pregnant. BMI (weight in kg/height in metres^2^) is based on estimated weight when measured weight exceeds a threshold of 130 kg.

In addition to BMI we also considered sociodemographic characteristics which may have an independent association with need for help with social care (sex, age, ethnicity, index of multiple deprivation (IMD) quintile, equivalised household income tertile (the tertile of household income after adjustment for household size), marital status, number of adults in the household, number of children in the household); and a binary variable indicating the presence of any limiting long term illness including mental illness.

The causal link between raised BMI and some long term illnesses leads to statistical difficulties in separating their effects on need for social care [6, 8]. In order to refine our estimates of the need that is ultimately attributable to BMI we constructed two additional measures of limiting long term illness and consider these separately in statistical analysis. One of the additional measures does not count individuals with only diabetes in its definition of long term illness, while the other does not count individuals with either diabetes or stroke, heart attack or angina. Both of these conditions are linked to raised BMI and the additional variables will allow the effect of other long term illnesses on need for social care to be more precisely identified. HSE does not distinguish between types of diabetes but over 90% of diabetes cases are type 2 [16], and of these approximately 79% to 83% are attributable to obesity and overweight [17, 18]. Approximately 34%–58% of cases of heart disease or stroke are attributable to overweight or obesity [17]. Other diseases are also linked to raised BMI but to a lesser extent, and as they also tend to occur less frequently in our sample we did not attempt to adjust for them.

We pooled data from the 2011, 2012 and 2013 HSE [19–21] in order to maximise sample size.

### Statistical analysis

The number reporting a need for help with social care in the HSE is higher than the number reporting that they received help with care [15]. This suggests an unmet need for support, with the determinants of need for care differing from the determinants of amount of help actually received. Consequently we adopted a two-step approach. In the first step we model the probability of need for care. In the second step we combine the model-predicted marginal probability of need for care by BMI with the overall mean hours of help received, conditional on receiving any help, to calculate the expected hours of help received per adult per week by unit of BMI. Thus our base case estimates of expected hours of social care received assume that there is no unmet need for care, that is care is being provided if there is a need, and that the reported hours provided are adequate to meet that need. We considered a respondent to have a self-reported need for care if they expressed at least difficulty with carrying out at least one ADL or IADL. This is consistent with the definition of need used in other HSE analyses [15].

The variables for hours of help received in the previous week are categorical for all sources of care in HSE 2011 and 2012, and for informal sources of care in HSE 2013. The categories used are: less than 1 h; 1–4 h; 5–9 h; 10–19 h; 20–34 h; 50–99 h; and (in HSE 2013 only) 100 h or more. We recoded these to numeric values using the midpoints of the categories. A value of 100 h was used for the category 100 h or more.

We estimated univariable and three multivariable logistic regression models to assess the association between the self-reported need for social care and its potential determinants. Multivariable model 1 controls for all covariates except those related to limiting long term illness; model 2 controls for all model 1 covariates plus any limiting long term illness; model 3 controls for all model 1 covariates plus the long term illness definition which excludes diabetes. A fourth multivariable model was used in sensitivity analysis and controls for all model 1 convariates plus the long term illness definition which excludes heart attack and stroke. The four models aim to provide progressively more refined estimation of the effect of BMI and its associated long term illnesses on the need for social care. Wald tests adjusted for use with survey data were used to assess statistical significance of the covariates.

The univariable models consider BMI as both a continuous and a categorical variable. In order fully to capture its association with need for social care, BMI enters the multivariable models as a continuous variable. To accommodate nonlinearities in this relation we included covariates for BMI, BMI squared (BMI^2^) and BMI cubed (BMI^3^). We recoded limiting long term illness to a dichotomous variable indicating the presence of limiting long term illness only. (Non-limiting long term illness was recoded as no limiting long term illness.) Missing values were coded as a separate category for categorical covariates.

Missing BMI was calculated after imputation of missing height and weight. We considered that imputation was necessary because the reasons for missing height and weight (individuals unable to stand or unsteady on their feet) are likely to be related to the need for social care. In these circumstances a complete case analysis would be biased [22]. Multiple imputation also permits a statistically more powerful analysis compared with analysis based on complete cases, as a greater number of observations may be included in modelling. A complete case analysis of model 3 was carried out as a sensitivity check.

Missing height and weight were imputed from all of the covariates considered in the regression models (except BMI) using multivariate normal multiple imputation with 50 imputations. Multivariate normal imputation was used as missing height and weight were assumed to be missing at random. Work elsewhere has indicated that this is a reasonable assumption with HSE data [23, 24]. As a validity check summary BMI statistics were calculated for a reduced dataset which excluded observations with missing BMI.

Analyses were undertaken in Stata 13.1 [25] and account for the HSE complex survey design and sample weights.

## Results

A sample of 6462 adults aged 65 or over was available for analysis after coding of missing values and imputation of missing height and weight. Excluding unreliable measurements 16% of the sample was missing both height and weight and 2% was missing either height or weight. For limiting long term illness there were missing data for 6 participants and these participants were excluded from analysis. Only one participant in HSE 2013 reported receipt of more than 100 h of formal help in the previous week from a single source. This observation is retained in the main analysis but its influence is considered in sensitivity analysis (Additional file 1: Table S1). It is worth noting that receipt of more than 100 h of care per week from informal sources is not unusual.

Characteristics of the study population are presented in Table 2. Those with a healthy non-missing BMI comprise 20.8% of the sample but represent only 15.0% of those with a self-reported need for social care while participants with any limiting long-term illness comprise 44.3% of the sample but make up 81.4% of those with a need for care. The effect of long term illness on need for care is proportionately and progressively slightly reduced if diabetes and then heart-related conditions are excluded from its definition, but remains pronounced. Females, older age groups, more deprived IMD quintiles, those who are not married or cohabiting and households with only one adult also have a need for care that is higher than their proportionate sample size.

**Table 2:** Sample characteristics and self-reported need for social care by sample characteristics

	Unweighted count	% or mean^a^	% of those with self-reported need for social care (95% CI)^a^
BMI (excludes missing and unreliable)	5045	28.06	
BMI (including unreliable and imputed)	6462	28.15	
BMI category			
< 18.5	52	0.8	1.1 (0.7 to 1.5)
18.5 to <25	1328	20.8	15.0 (13.4 to 16.6)
25 to <30	2152	33.2	23.0 (21.2 to 24.8)
30 to <40	1405	21.7	22.9 (21.1 to 24.7)
40+	108	1.7	2.5 (1.8 to 3.1)
Missing and unreliable	1417	21.8	35.6 (33.3 to 37.8)
Limiting long term illness			
No	3632	55.7	18.6 (16.9 to 20.2)
Yes	2830	44.3	81.4 (79.8 to 83.1)
Limiting illness (definition excludes any diabetes)			
No	4098	63.0	33.3 (31.3 to 35.3)
Yes	2364	37.0	66.7 (64.7 to 68.7)
Limiting illness (definition excludes any diabetes or heart-related/stroke)			
No	4398	67.7	43.2 (41.0 to 45.3)
Yes	2064	32.3	56.8 (54.7 to 59.0)
Sex			
Female	3500	45.3	62.6 (60.8 to 64.3)
Male	2962	54.7	37.4 (35.7 to 39.2)
Age (years)			
65–69	2004	30.4	17.2 (15.5 to 18.9)
70–74	1609	24.3	19.6 (17.9 to 21.3)
75–79	1262	20.0	19.9 (18.1 to 21.7)
80–84	904	14.4	21.9 (20.2 to 23.7)
85+	683	11.0	21.4 (19.6 to 23.2)
Ethnic Origin			
White	6202	95.6	94.5 (93.4 to 95.7)
Asian	126	2.2	3.1 (2.2 to 4.0)
Black	77	1.3	1.3 (0.8 to 1.9)
Other	31	0.5	0.5 (0.2 to 0.8)
Missing	26	0.4	0.5 (0.2 to 0.8)
IMD quintile			
1 least deprived	1474	22.6	16.9 (14.9 to 19.0)
2	1592	24.9	22.3 (20.1 to 24.4)
3	1405	21.9	21.3 (19.2 to 23.3)
4	1105	17.1	19.9 (17.8 to 21.9)
5 most deprived	886	13.4	19.6 (17.5 to 21.8)
Equivalised income tertile			
Lowest	1994	30.9	36.1 (34.0 to 38.3)
Middle	1867	28.7	26.0 (24.0 to 28.0)
Highest	860	13.3	7.2 (6.0 to 8.3)
Missing	1741	27.1	30.7 (28.6 to 32.9)
Marital status			
Not married or cohabiting	2566	40.3	53.0 (50.7 to 55.4)
Married or cohabiting	3896	59.7	47.0 (44.6 to 49.3)
Adults in household			
1	2291	35.4	45.6 (43.3 to 47.8)
2	3746	56.6	45.6 (43.3 to 47.9)
3	352	6.5	6.7 (5.4 to 8.0)
4	55	1.1	1.5 (0.8 to 2.1)
5	10	0.2	0.2 (−0.1 to 0.6)
6	6	0.1	0.3 (0.0 to 0.6)
8	2	0.1	0.2 (0.2 to 0.2)
Children in household			
0	6401	98.9	98.3 (97.7 to 98.9)
1	38	0.7	1.2 (0.7 to 1.7)
2	18	0.3	0.4 (0.1 to 0.7)
3	3	0.1	0.0 (0.0 to 0.0)
4	1	0.0	0.1 (−0.1 to 0.2)
5	1	0.0	0.0 (0.0 to 0.1)
Any help required with ADL or IADL			
No	4194	64.3	
Yes	2268	35.7	
Any help received with ADL or IADL in the previous week			
No	5024	77.3	
Yes	1438	22.7	
Type of care received in the previous week^b^			
None	5024	77.3	
Informal only	1222	19.2	
Formal (LA) only	78	1.3	
Both informal and formal	138	2.2	
Mean hours of help received by source in previous week^b^			
Informal	1330	21.2	
Formal (LA)	214	1.6	
Total informal and formal	1406	22.8	

^a^ Weighted and adjusted for survey design

^b^Conditioned on receiving at least some help and expressing a need for help

Table 2 shows that the mean BMI after imputation is within one tenth of a unit of the mean BMI calculated excluding missing or unreliable measurements. This is likely to reflect the relatively small proportion of the group for whom imputations were made but indicates that imputation is satisfactory under the missing at random assumption. Those with a missing or unreliable BMI measurement are more likely to require social care than healthy or overweight BMI categories, as indicated by the categorical BMI variable in Table 2.

Table 3 gives the odds ratios estimated by the logistic regression models. All variables are significant at the 95% level in the unadjusted models. However the number of children in a household is not a significant predictor of self-reported need for social care in multivariable models 1 or 3 while the number of adults in a household is not significant at the 95% level in models 2 or 3. A cubic functional form was found to provide the best fit between BMI and the observed data in all three multivariable models.

**Table 3:** Results of logistic regressions exploring the association between self-reported need for social care and sociodemographic characteristics. *n* = 6462 in all models^a^

	Unadjusted Odds Ratio (95% CI)	Adjusted Odds Ratios Model 1 (95% CI)	Adjusted Odds Ratios Model 2 (95% CI)	Adjusted Odds Ratios Model 3 (95% CI)
BMI (excludes missing)	1.05 (1.04 to 1.07)**			
BMI	1.05 (1.04 to 1.07)**	0.36 (0.21 to 0.62)**	0.48 (0.27 to 0.85)*	0.44 (0.25 to 0.78)**
BMI^2^		1.03 (1.02 to 1.05)**	1.02 (1.00 to 1.04)*	1.03 (1.01 to 1.05)**
BMI^3^		1.00 (1.00 to 1.00)**	1.00 (1.00 to 1.00)*	1.00 (1.00 to 1.00)*
BMI category				
< 18.5	2.61 (0.22 to 0.67)**			
18.5 to <25	Ref			
25 to <30	0.95 (0.80 to 1.12)			
30 to <40	1.75 (1.47 to 2.08)**			
40+	3.19 (2.13 to 4.77)**			
Missing	4.03 (3.38 to 4.81)**			
Limiting long term illness				
No	Ref		Ref	
Yes	14.18 (12.40 to 16.21)**		12.70 (10.99 to 14.68)**	
Limiting long term illness excluding any diabetes				
No	Ref			Ref
Yes	7.81 (6.93 to 8.79)**			7.21 (6.34 to 8.21)**
Limiting illness excluding any diabetes or heart-related/stroke				
No	Ref			
Yes	5.73 (5.10 to 6.45)**			
Sex				
Female	Ref	Ref	Ref	Ref
Male	0.61 (0.55 to 0.67)**	0.70 (0.63 to 0.79)**	0.70 (0.61 to 0.80)**	0.74 (0.65 to 0.84)**
Age (years)				
65–69	Ref	Ref	Ref	Ref
70–74	1.60 (1.37 to 1.88)**	1.60 (1.35 to 1.88)	1.50 (1.25 to 1.81)**	1.56 (1.31 to 1.86)**
75–79	2.18 (1.83 to 2.59)**	2.10 (1.75 to 2.51)**	1.89 (1.54 to 2.31)**	1.99 (1.64 to 2.41)**
80–84	4.71 (3.94 to 5.64)**	5.03 (4.15 to 6.09)**	3.99 (3.16 to 5.03)**	4.30 (3.47 to 5.34)**
85+	9.08 (7.42 to 11.11)**	9.89 (7.92 to 12.36)**	9.29 (7.21 to 11.98)**	9.17 (7.24 to 11.63)**
Ethnic Origin				
White	Ref	Ref	Ref	Ref
Asian	1.81 (1.18 to 2.76)**	1.96 (1.23 to 3.12)**	1.81 (1.04 to 3.15)*	2.70 (1.56 to 4.67)**
Black	1.05 (0.65 to 1.72)	0.66 (0.39 to 1.10)	0.70 (0.42 to 1.16)	0.80 (0.49 to 1.31)
Other	1.09 (0.54 to 2.17)	1.26 (0.62 to 2.57)	1.38 (0.49 to 3.92)	1.41 (0.51 to 3.93)
Missing	1.91 (0.92 to 3.96)	1.19 (0.50 to 2.83)	1.20 (0.50 to 2.86)	1.38 (0.61 to 3.17)
IMD quintile				
1 least deprived	Ref	Ref	Ref	Ref
2	1.29 (1.09 to 1.51)**	1.11 (0.93 to 1.33)	1.08 (0.87 to 1.32)	1.11 (0.91 to 1.35)
3	1.45 (1.22 to 1.72)**	1.28 (1.07 to 1.53)**	1.10 (0.90 to 1.35)	1.17 (0.96 to 1.43)
4	1.94 (1.62 to 2.31)**	1.55 (1.27 to 1.88)**	1.35 (1.09 to 1.67)**	1.46 (1.18 to 1.79)**
5 most deprived	3.00 (2.47 to 3.63)**	2.42 (1.94 to 3.03)**	2.02 (1.58 to 2.58)**	2.22 (1.76 to 2.81)**
Equivalised income tertile				
Lowest	Ref	Ref	Ref	Ref
Middle	0.67 (0.58 to 0.77)**	0.81 (0.69 to 0.95)*	0.88 (0.73 to 1.05)	0.83 (0.70 to 0.99)*
Highest	0.33 (0.27 to 0.41)**	0.49 (0.39 to 0.61)**	0.62 (0.48 to 0.81)**	0.56 (0.44 to 0.71)**
Missing	0.95 (0.82 to 1.09)	0.86 (0.74 to 1.01)	0.97 (0.81 to 1.16)	0.94 (0.79 to 1.11)
Marital status				
Not married or cohabiting	Ref	Ref	Ref	Ref
Married or cohabiting	0.44 (0.39 to 0.49)**	0.67 (0.55 to 0.82)**	0.69 (0.56 to 0.86)**	0.68 (0.55 to 0.83)**
Adults in household	0.75 (0.68 to 0.84)**	1.19 (1.02 to 1.38)*	1.06 (0.90 to 1.24)	1.09 (0.94 to 1.27)
Children in household	1.56 (1.12 to 2.19)**	1.24 (0.86 to 1.78)	1.58 (1.09 to 2.27)*	1.35 (0.96 to 1.89)

^a^ Model 2 is adjusted for limiting long term illness, in addition to the sociodemographic characteristics also considered in model 1; model 3 is the same as model 2 but uses a definition of long term illness which does not include diabetes

** *p* < 0.01, * *p* < 0.05

In model 1 a self-reported need for social care is positively associated with female sex; age; Asian ethnic origin; more deprived IMD quintiles; lower equivalised income tertiles; and unmarried status. In model 2 these positive relations remain but are generally less pronounced. For example the average odds of need for social care in the 80–84 age group are 5.03 times those in the reference age group 65–69 in model 1 (95% CI 4.15 to 6.09) but are 3.99 times those of age 65–69 in model 2 (95% CI 3.16 to 5.03). Model 2 shows that an individual with a limiting long-term illness has odds of a self-reported need for social care which are 12.70 times those of people without a limiting long term illness (95% CI 10.99 to 14.68). This compares with the odds ratio of 14.18 seen in the univariable model (95% CI 12.40 to 16.21). The limiting illness odds ratios estimated in model 3, which excludes diabetes from its definition, are midway between these two estimates.

Regression models estimated without imputed BMI observations do not show any substantive deviation in significance or direction of the relation between BMI and social care need but do show a reduced magnitude of the relation (Additional file 1: Fig. S1). This indicates that missing height and weight is as anticipated related to the need for social care (also confirmed by Table 2) and that a complete case analysis is consequently biased.

**Fig. 1:**
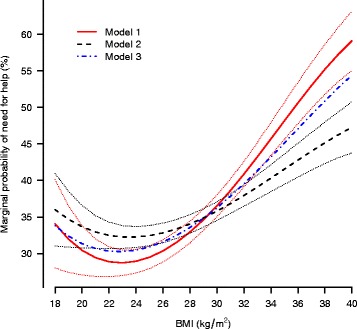
Marginal predicted probabilities of self-reported need for help with Activities of Daily Living (ADL) and Instrumental Activities of Daily Living (IADL), by BMI. Dotted lines represent 95% confidence intervals. Model 2 is adjusted for limiting long term illness, in addition to the sociodemographic characteristics also considered in model 1. Model 3 is the same as model 2 but uses a definition of long term illness which does not include diabetes

Figure 1 compares the marginal probabilities of self-reported need for social care by BMI obtained from models 1 to 3. These probabilities were calculated as the average probability of need for social care of the sample at various values of BMI. The best-fit relation between BMI and need for social care is J-shaped in all models over the range of BMI considered, but the need for social care increases more rapidly with BMI under model 1 than models 2 or 3 as model 1 reflects the association of BMI and long term illness as well as the physical and social difficulties it can entail. For example an increase in BMI from 29 to 30 is associated with a marginal increase in probability of need for care of 1.91% in model 1 but 0.93% in model 2.

The predicted probabilities of self-reported need for care shown in Fig. 1 may be combined with the average hours of care received from Table 2 to calculate expected hours of help received by BMI if needs were to be met with the current average provision per week. Table 2 shows that, conditional on receiving at least some help and expressing a need for help, the mean hours of care received per week is 21.2 h from informal sources and 1.6 from formal local authority sources. Table 4 gives the expected hours of care received by BMI for both formal and informal sources calculated by combining these estimates and the model marginal probabilities of need for help from Fig. 1. An alternative version of Table 4 excluding one individual in receipt of more than 100 h of formal care per week is provided in Additional file 1 (Table S1).

**Table 4:** Expected hours of community-based social care per person per week by source of care and BMI in population aged 65 and over. Assumes that current mean hours of help received is spread uniformly across the BMI distribution

BMI	Model 1		Model 2		Model 3	
	Formal local authority	Informal	Formal local authority	Informal	Formal local authority	Informal
18	0.54	7.22	0.58	7.63	0.54	7.16
19	0.51	6.78	0.55	7.35	0.52	6.86
20	0.49	6.45	0.54	7.14	0.50	6.65
21	0.47	6.24	0.53	6.99	0.49	6.50
22	0.46	6.12	0.52	6.89	0.49	6.43
23	0.46	6.09	0.52	6.84	0.48	6.42
24	0.46	6.14	0.52	6.84	0.49	6.46
25	0.47	6.25	0.52	6.88	0.49	6.55
26	0.49	6.44	0.53	6.96	0.51	6.70
27	0.50	6.68	0.53	7.07	0.52	6.89
28	0.53	6.99	0.54	7.22	0.54	7.12
29	0.55	7.34	0.56	7.39	0.56	7.39
30	0.58	7.74	0.57	7.59	0.58	7.69
31	0.62	8.19	0.59	7.81	0.61	8.02
32	0.65	8.67	0.61	8.04	0.63	8.38
33	0.69	9.17	0.63	8.29	0.66	8.76
34	0.73	9.69	0.65	8.55	0.69	9.16
35	0.77	10.21	0.66	8.81	0.72	9.57
36	0.81	10.73	0.68	9.07	0.75	9.98
37	0.85	11.23	0.70	9.33	0.78	10.39
38	0.88	11.70	0.72	9.57	0.81	10.79
39	0.92	12.14	0.74	9.81	0.84	11.17
40	0.95	12.53	0.76	10.02	0.87	11.53

## Discussion

Our findings show that BMI is positively associated with self-reported need for social care after adjustment for sociodemographic characteristics and limiting long term illness. This agrees with previous studies which have found that the association between overweight and obesity and health or allied health service use is only partially explained by chronic health conditions [8, 13]. The adjusted BMI odds ratio estimates from model 2 can be interpreted as reflecting the physical and social difficulties of obesity [2]. The adjusted BMI estimates from model 1 reflect implicitly, in addition, the association of BMI with all limiting long term conditions while the adjusted estimates from model 3 reflect the association of BMI with limiting long term diabetes only.

Comparison of the BMI and social care need relation of model 1 with the relation found for model 2 (Fig. 1) indicates that BMIs under 30 are associated with a lower than average prevalence of long term illness since the marginal predicted need for social care is smaller in this BMI range under model 1 than model 2. Conversely BMIs above 30 are associated with a higher than average prevalence of long term illness because the marginal need for care is smaller in this BMI range when long-term illness is adjusted for, that is under model 2. These relations thus reflect the well documented adverse health consequences of obesity [1, 26].

J-shaped relations between BMI and risk of all cause mortality have been reported elsewhere [27–32] and have also been found for other risk exposures including alcohol consumption [33]. The lowest modelled probabilities of need of care are found at BMIs between approximately 21 and 25 (Fig. 1) and this range broadly corresponds to the optimum BMI range from a mortality perspective [32]. The left lip of the J-shaped curve for BMI and mortality reflects calorie malnutrition [33] and this is a plausible explanation of the upturn in need for social care at BMIs less than 21. However further work is required to confirm the causal mechanisms.

Only a proportion of the long term conditions considered here are related to obesity, and of these only a fraction is attributable to obesity [18]. Model 1 captures the association of BMI with long term conditions but does not distinguish which long term conditions are caused by high BMI, or conversely whether high BMI was caused by a long term illness. On the other hand model 2 removes any long term illness effect of BMI, and is thus overly conservative in associating social care need with BMI. Model 3 considers that diabetes is largely explained by BMI and indicates that the effect of BMI on need for social care lies somewhere between the estimates given by model 1 and model 2. Results for sensitivity analysis model 4, which considers that both diabetes and heart-related conditions are largely explained by BMI, lie between the estimates given by model 1 and model 3, though closer to those of model 3. The estimates of model 3 are thus more pragmatic than the estimates given by models 1 and 2 but a prudent approach in economic modelling would be to examine the effect of all predicted sets of probability of care need on cost-effectiveness of interventions.

The expected hours of help received (Table 4) can be combined with the average hourly cost of a care worker to provide base case estimates of the cost of social care by BMI for use in economic models. The total cost of providing a home care worker in England is approximately £24 per hour [34] and on this basis, using model 3, the annual cost of local authority funded community-based social care for an individual with a BMI of 40 would be £1086 (£24*52 weeks * 0.87 h per week (Table 4)). The same cost for an individual with a BMI of 23 (ie, within the healthy range for BMI) is £599. For a typical English lower tier local authority with a population of 30,000 adults aged over 65 [35] and a morbid obesity prevalence of 2.9% [36] this equates to an annual excess social care cost of £423,000. These estimates assume that provision of social care will continue at current costs in the short term as obesity is only one of many factors driving social care utilisation.

These base case estimates for formal care costs are non-conservative because they assume that all those who indicate a need for care are in receipt of at least some care. However Table 2 demonstrates that there is a large unmet need for care as only 1438 respondents reported receiving help in the last week, while 2268 expressed a need for help. This equates to 62% of the weighted sample, i.e. 38% of those who expressed a need for help with at least one ADL or IADL did not receive any help. The base case cost estimates could be reduced to reflect the unmet need for care, and provide more conservative estimates for scenario analysis. Alternatively a less conservative scenario might consider that current formal social care provision is not sufficient and increase the assumed hours received per person per week, thereby increasing the cost. The wider societal cost of informal social care provision from friends and family members might also be included if an economic model is to reflect a societal cost perspective.

We opted to produce a model of the self-reported need for social care rather than directly model the hours of formal local authority help received because there were only 214 individuals in our sample of 6462 who both needed and received formal local authority care and we did not feel that this was sufficient robustly to model the hours of care received by BMI while also adjusting for other factors. Previous studies have not found a significant association between BMI category and use of home help services which may be due to a limited sample as typically only a small proportion of adults receives state-provided home help [8, 13]. Given that help needed and help received are governed by two separate processes a two-part model is also likely to be required [3]. For these reasons we used the unadjusted mean hours of help received to calculate expected hours of help received (Table 4). This is likely to be conservative as the mean hours of local authority help received may increase with BMI.

### Strengths and limitations of the study

Our analyses use a large probabilistic sample from HSE and are therefore reasonably robust and nationally representative. Height and weight are objectively measured at interview in HSE and not subject to self-report bias. We have made conservative assumptions and used a transparent method which provides a basis for further work.

Our study is cross-sectional in design and as such we are not able to infer causation. The findings indicate that BMI is positively associated with social care need but it is possible that social care need precedes raised BMI in time, rather than the other way around. Further work might include analysis of longitudinal data which would help to inform this question. As a cross-sectional study the estimates presented here measure the association of BMI with social care need. This may be an overestimate of causal effect if receipt of social care gives rise to higher BMIs, or an underestimate if receipt of social care gives rise to lower BMIs. However this potential endogeneity is mitigated as we have modelled need for care rather than receipt of care: given that a proportion of those needing care do not receive it (Table 2) any impact of social care receipt on BMI status is reduced.

HSE does not cover people living in care homes. The care home population is likely to be on average older and less healthy, and thus have a higher level of social care need, than the population in private households [15]. If the care home population is on average more obese than the population in private households then our estimates of the effect of BMI on need for social care are biased downwards.

Questions about social care are only put to HSE participants aged 65 and older. Although adults of this age represent 63% of those in receipt of community-based social care [15] this is nonetheless a limitation from an economic model point of view where estimates of social care use by BMI are required for all age groups. Assumed or extrapolated age adjustments will need to be applied to the marginal probability estimates in order to reflect the need for social care in younger age groups.

We have used BMI as a measure of obesity. Consideration of alternative measures of obesity such as waist-to-height ratio may provide a more thorough understanding of the association between social care need and weight status [7, 37].

## Conclusions

BMI is positively associated with self-reported need for social care after adjustment for sociodemographic factors and limiting long-term illness. The increase in need for care with BMI gives rise to additional costs in social care provision which should be borne in mind when calculating the cost-effectiveness of interventions aimed at reducing obesity.

We have incorporated our estimates into subsequent versions of the PHE weight management cost-effectiveness tool in order to improve the usefulness of this tool for estimating the potential benefits of interventions and broader activities to reduce the prevalence of obesity. It will be important to obtain better data and research evidence in future in order both to refine our estimates of the social care costs associated with obesity and to provide a fuller picture of the relation between weight status and social care costs in the care home population and at younger ages.

### Acknowlegements

We are grateful to the consultees of version 1 of the economic assessment tool and to Lesley Manning of Buckinghamshire County Council for providing the user feedback which prompted this work. We would also like to thank the reviewers for their comments which have greatly improved the paper.

## Additional file

Additional file 1:
**Fig. S1.** Marginal predicted probabilities of self-reported need for help with Activities of Daily Living (ADL) and Instrumental Activities of Daily Living (IADL), by BMI. Dotted lines represent 95% confidence intervals. Model 3 is adjusted for limiting long term illness, in addition to sociodemographic characteristics. Its definition of long term illness does not include diabetes. Model 3 uses BMI calculated from multiply imputed height and weight (*n* = 6462). Model 3CC is the same as model 3 but based on a complete case analysis – observations with missing height and weight are not included (*n* = 5045). **Table S1.** Expected hours of community-based social care per person per week by source of care and BMI in population aged 65 and over. Sensitivity analysis using mean hours of help calculated after removal of one outlying observation of more than 100 h of formal care per week. (DOCX 106 kb)

## Abbreviations

ADL: Activities of Daily Living
BMI: Body mass index
HSE: Health Survey for England
IADL: Instrumental Activities of Daily Living
IMD: Index of multiple deprivation
PHE: Public Health England
