# Supplementary material for: Estimating the variation in need for community-based social care by body mass index in England and associated cost: population-based cross-sectional study
Source: BMC Public Health. 2017 Aug 22;17:667. doi: 10.1186/s12889-017-4665-1 (PMC5567467; doi:10.1186/s12889-017-4665-1)
Supplement: Additional file 1: — Fig. S1. Marginal predicted probabilities of self-reported need for help with Activities of Daily Living (ADL) and Instrumental Activities of Daily Living (IADL), by BMI. Dotted lines represent 95% confidence intervals. Model 3 is adjusted for limiting long term illness, in addition to sociodemographic characteristics. Its definition of long term illness does not include diabetes. Model 3 uses BMI calculated from multiply imputed height and weight (n = 6462). Model 3CC is the same as model 3 but based on a complete case analysis – observations with missing height and weight are not included (n = 5045). Table S1. Expected hours of community-based social care per person per week by source of care and BMI in population aged 65 and over. Sensitivity analysis using mean hours of help calculated after removal of one outlying observation of more than 100 h of formal care per week. (DOCX 106 kb) [file 12889_2017_4665_MOESM1_ESM.docx]

Additional file 1

**Estimating the variation in need for community-based social care by body mass index in England and associated cost: population-based cross-sectional study**

Vicky R Copley, Nick Cavill, Jane Wolstenholme, Richard Fordham, Harry Rutter

Figure S1. Marginal predicted probabilities of self-reported need for help with Activities of Daily Living (ADL) and Instrumental Activities of Daily Living (IADL), by BMI. Dotted lines represent 95% confidence intervals. Model 3 is adjusted for limiting long term illness, in addition to sociodemographic characteristics. Its definition of long term illness does not include diabetes. Model 3 uses BMI calculated from multiply imputed height and weight (n=6,462). Model 3CC is the same as model 3 but based on a complete case analysis – observations with missing height and weight are not included (n=5,045).


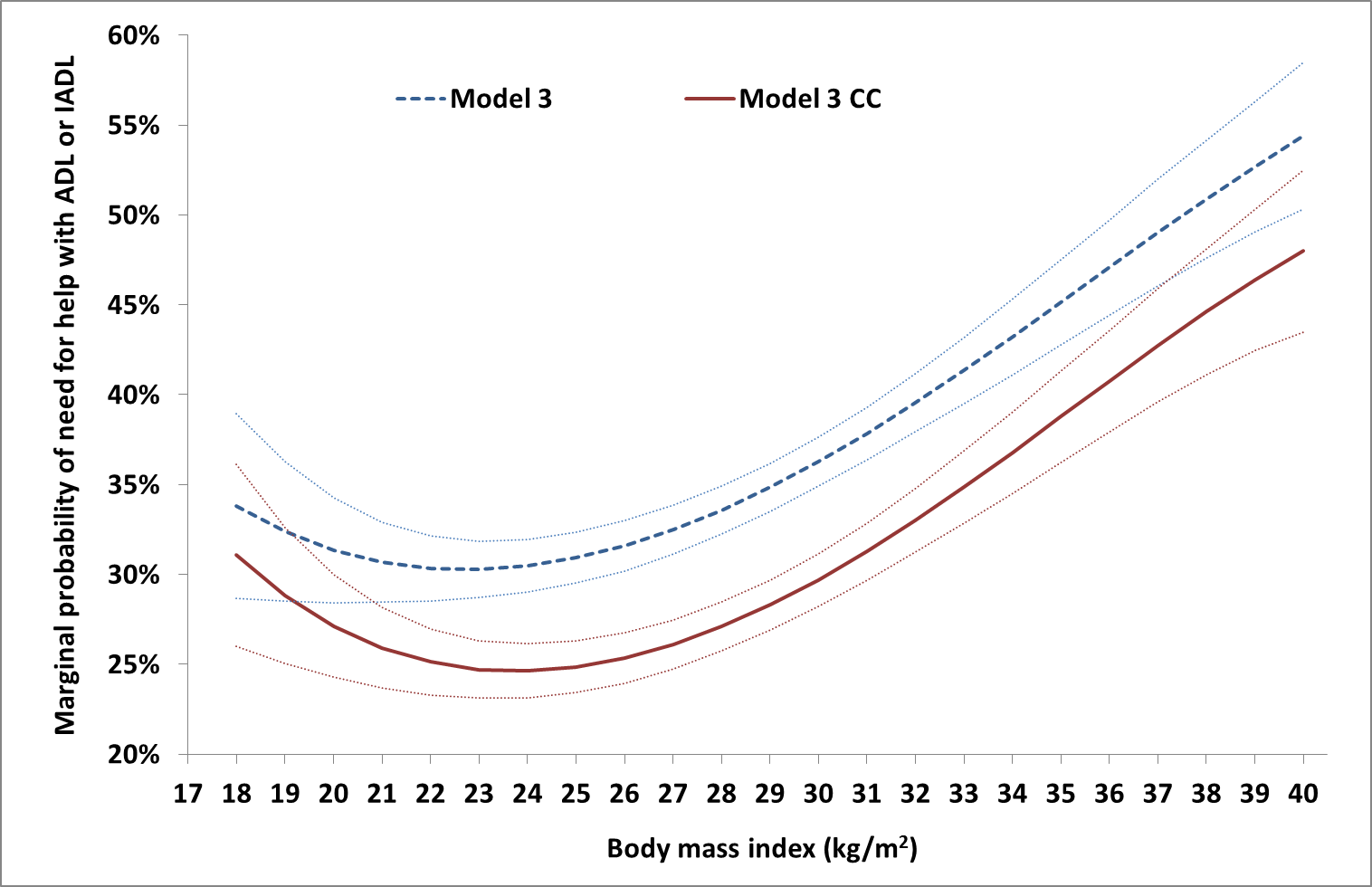


Table S1. Expected hours of community-based social care per person per week by source of care and BMI in population aged 65 and over. Sensitivity analysis using mean hours of help calculated after removal of one outlying observation of more than 100 hours of formal care per week.

|  | Model 1 |  |  | Model 2 |  |  | Model 3 |  |
| --- | --- | --- | --- | --- | --- | --- | --- | --- |
| BMI | Formal local authority | Informal |  | Formal local authority | Informal |  | Formal local authority | Informal |
| 18 | 0.50 | 7.22 |  | 0.53 | 7.63 |  | 0.49 | 7.16 |
| 19 | 0.47 | 6.78 |  | 0.51 | 7.35 |  | 0.47 | 6.86 |
| 20 | 0.44 | 6.45 |  | 0.49 | 7.14 |  | 0.46 | 6.65 |
| 21 | 0.43 | 6.24 |  | 0.48 | 6.99 |  | 0.45 | 6.50 |
| 22 | 0.42 | 6.12 |  | 0.47 | 6.89 |  | 0.44 | 6.43 |
| 23 | 0.42 | 6.09 |  | 0.47 | 6.84 |  | 0.44 | 6.42 |
| 24 | 0.42 | 6.14 |  | 0.47 | 6.84 |  | 0.44 | 6.46 |
| 25 | 0.43 | 6.25 |  | 0.47 | 6.88 |  | 0.45 | 6.55 |
| 26 | 0.44 | 6.44 |  | 0.48 | 6.96 |  | 0.46 | 6.70 |
| 27 | 0.46 | 6.68 |  | 0.49 | 7.07 |  | 0.47 | 6.89 |
| 28 | 0.48 | 6.99 |  | 0.50 | 7.22 |  | 0.49 | 7.12 |
| 29 | 0.51 | 7.34 |  | 0.51 | 7.39 |  | 0.51 | 7.39 |
| 30 | 0.53 | 7.74 |  | 0.52 | 7.59 |  | 0.53 | 7.69 |
| 31 | 0.56 | 8.19 |  | 0.54 | 7.81 |  | 0.55 | 8.02 |
| 32 | 0.60 | 8.67 |  | 0.55 | 8.04 |  | 0.58 | 8.38 |
| 33 | 0.63 | 9.17 |  | 0.57 | 8.29 |  | 0.60 | 8.76 |
| 34 | 0.67 | 9.69 |  | 0.59 | 8.55 |  | 0.63 | 9.16 |
| 35 | 0.70 | 10.21 |  | 0.61 | 8.81 |  | 0.66 | 9.57 |
| 36 | 0.74 | 10.73 |  | 0.62 | 9.07 |  | 0.69 | 9.98 |
| 37 | 0.77 | 11.23 |  | 0.64 | 9.33 |  | 0.72 | 10.39 |
| 38 | 0.81 | 11.70 |  | 0.66 | 9.57 |  | 0.74 | 10.79 |
| 39 | 0.84 | 12.14 |  | 0.68 | 9.81 |  | 0.77 | 11.17 |
| 40 | 0.86 | 12.53 |  | 0.69 | 10.02 |  | 0.79 | 11.53 |
